# Symmetrical Peripheral Gangrene Associated with Low Output Cardiac Failure †

**DOI:** 10.3390/medicina55070383

**Published:** 2019-07-17

**Authors:** Sijan Basnet, Priya Rajagopalan, Rashmi Dhital, Ataul Qureshi

**Affiliations:** 1 Department of Medicine, Reading Hospital and Medical Center, West Reading, PA 19611, USA; 2 Thomas Jefferson University Hospital, 1025 Walnut Street, Philadelphia, PA 19107, USA

**Keywords:** symmetrical peripheral gangrene, heart failure, low flow state

## Abstract

Symmetrical peripheral gangrene (SPG) is a rare entity characterized by ischemic changes of the distal extremities with maintained vascular integrity. We present the case of a 64-year-old man with bilateral necrotic toes and deranged liver function tests. This was thought to be related to severely depressed ejection fraction from non-ischemic etiology, presumably chronic alcohol ingestion. We hope that awareness of SPG and association with a low output state will aid in early detection and prevention.

## 1. Introduction

Symmetrical peripheral gangrene (SPG) was first described by Hutchison in 1891 [1]. SPG is a rare clinical condition with acute, symmetrical ischemia of two or more extremities, leading to gangrene in the absence of large vessel obstruction or vasculitis [2,3,4,5]. Various infective and noninfective factors are responsible for the development of SPG [3]. Among them, cardiac conditions myocardial infarction [6,7,8], myocardial infarction with coronary artery bypass graft and, such as ventricular septal defect repair [9], paroxysmal ventricular tachycardia [10], ventricular pseudoaneurysm [11], and severe heart failure [12] have been associated with SPG. We present a case of SPG in the setting of severely depressed cardiac ejection fraction.

## 2. Case Description

Our patient is a 64-year-old man referred to our emergency department from an outside hospital for management of liver failure and bilateral necrotic toes. The patient was confused at the time of presentation and was not able to provide a detailed history. He was accompanied by his niece, who explained that the patient had had intermittent episodes of confusion over the past several days prior to presentation. He was taken to the referring hospital because his niece had noticed discoloration of his toes and progressive yellowing of the skin. As the patient’s mentation got better during the hospital stay, he mentioned that he had had nausea, vomiting, diarrhea, and abdominal pain for three months prior to admission. He does not remember being out in the cold. He denied recent medications, including antibiotics, herbal supplement use, and drug use, and he also denied recent travel and tick bites. He did not have any urinary complaints. He did not have dyspnea, orthopnea, chest pain, a change in exercise tolerance, or pedal edema. His past medical history was significant due to a motor vehicle accident with a traumatic brain injury, complex regional pain syndrome, a prostate carcinoma status post-transurethral resection of the prostate, depression, and hypertension. He was never told he had liver or heart problems. His home medications were aspirin, hydrochlorothiazide, irbesartan, duloxetine, gabapentin, clonazepam, and tramadol. He smoked a few cigarettes per day but had been up to a pack per day for 4–5 years in total alongside marijuana use. Upon questioning, he stated that he drank 3–4 beers and 2–3 glasses of liquor daily. Later, his son mentioned that he had found multiple empty bottles of liquor prior to admission. The patient lived independently in his apartment and was able to take care of himself. He reported good exercise tolerance without any anginal symptoms.

**Figure 1:**
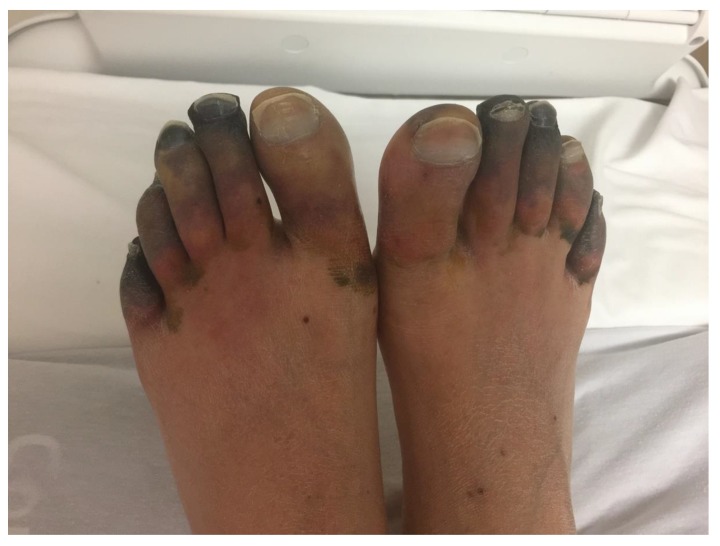
Bilateral gangrenous toes.

At presentation, his temperature was 98 °F (36.7 °C), his pulse was 103 beats per min, his blood pressure was 107/84 mm Hg, and his respiratory rate was 17 per min, maintaining saturation at 96% on room air. He was icteric on examination. He had sparse bibasilar rales on chest examination. Cardiac examination was suggestive of parasternal heave with an enlarged point of maximal impulse. He had dry and gangrenous second through fifth toes and densely cyanotic first toes, bilaterally (Figure 1). This subsequently progressed to necrosis of all his toes. He did not have any other skin or mucous membrane changes. His bilateral dorsalis pedis and posterior tibialis pulses were palpable.

## 3. Cardiomyopathy

His electrocardiogram (EKG) showed sinus tachycardia with a left bundle branch block with occasional premature ventricular complexes. Transthoracic echocardiography showed an ejection fraction of 10% with global hypokinesis and severe left ventricular enlargement but no embolus. His brain-type natriuretic peptide (BNP) was 18,984 pg/mL (reference range: 0–100 pg/mL). Troponin and thyroid stimulating hormones were within normal ranges. A left heart catheterization showed non-obstructive coronary artery disease. A right heart catheterization showed elevated pulmonary artery pressures and filling pressures with a cardiac index of 1.28 L/min/sqm. The patient was started on dobutamine infusion for inotropic support. His cardiomyopathy was thought to be related to alcohol intake because the patient had been consuming moderate amounts of alcohol for more than 20 years. He was also put on lisinopril for afterload reduction. The patient improved clinically in the next five days and was weaned off dobutamine. He was not thought to be a candidate for a heart transplant or advanced therapies based on ongoing alcohol abuse or lack of social support.

## 4. Liver Failure

His liver function tests at the referring hospital were deranged (Table 1), which was eventually thought to be secondary to the reduced cardiac index with hypoperfusion of liver and chronic alcohol abuse. His liver function tests improved with clinical improvement and initiation of dobutamine. Computerized tomography (CT) of abdomen/pelvis was consistent with hepatic steatosis. Viral hepatitis panels, Tylenol levels, anti-mitochondrial antibodies, liver kidney microsomal antibodies, and anti-smooth muscle antibodies for autoimmune hepatitis, and ceruloplasmin for Wilson’s disease were negative (Table 1).

## 5. Necrotic Toes

His white count was slightly elevated at 12,500/µL (reference range: 4000–11,000/µL). His erythrocyte sedimentation rate (ESR) was 25 mm/h (reference range: 0–15 mm/h) and his C-reactive protein (CRP) was 6.20 mg/dL (reference range: ≤0.80 mg/dL). His ESR and CRP were believed to be elevated secondary to the gangrene. CT angiography of the chest/abdomen/pelvis showed patent aorta and branch vessels without significant stenosis. Doppler ultrasound of legs showed normal ankle-brachial index and ankle waveforms. Rheumatology was also consulted to rule out vasculitis as a potential cause of his presentation; however, his workup was negative. His necrosis was thought to be secondary to the decreased peripheral perfusion and redistribution to vital organs in the setting of acute heart failure. Skin biopsy was not done for concern for risk of infection. The patient was planned for an outpatient podiatry follow-up for possible amputation but was lost to the follow up.

## 6. Ethical Statement

Informed consent was obtained from the patient for submission of the case.

## 7. Discussion

### 7.1. Pathogenesis

The exact mechanism for SPG is unclear [3]. A hypercoagulable disseminated intravascular coagulation (DIC)-like condition secondary to sepsis has been associated in 85 to 100% of patients with SPG [4,11]. Our patient was not septic on presentation and did not have a clear source of infection that might have led to DIC. In the case of severe right heart failure, Fishberg et al. first noted engorged jugular veins and elevated jugular venous pressure with collapsed veins of extremities occurring reflexively to redistribute blood supply to essential organs, such as the brain [13]. With decreased peripheral perfusion pressures from 36 to 60 mm Hg, blood flow through digital arteries stops [2,14]. Low flow state combined with microvascular occlusion from DIC [3,15] leads to ischemic changes that involve distal extremities and advances, proximally [3]. Aggravating factors include cold-induced vasospasm [2,11], the use of vasoconstrictor drugs [2,3], diabetes mellitus, and renal failure [2].

### 7.2. Clinical Presentation

The exact incidence of SPG is not known [3]. Studies have reported both male [16] and female [17] preponderance. SPG initially presents with cold and pale extremities followed by dusky discoloration of the skin [2,3,4]. This progressively worsens to dry gangrene in 12–24 h with the formation of a line of demarcation in about two weeks. Peripheral pulses are palpable except in cases with the use of vasoconstrictor medications. Ear lobes, upper lip, scalp, genitalia, and the tip of the nose can be involved in severe cases [4]. Our patient had gangrenous involvement of only his toes.

### 7.3. Diagnosis

A diagnosis should be suspected in patients with bluish discoloration of extremities and elevated lactate levels. Presence of schistocytes on a peripheral smear may indicate microangiopathic changes from DIC. However, DIC may occur late, and repeated screening should be done. Doppler ultrasound of the extremities shows intact peripheral pulses [3,9]. Cohen et al. noted that sections taken from the left dorsalis pedis artery were non-occluded in patients with SPG [9]. Post-mortem examinations have also found occlusion of the small vessels [2].

### 7.4. Management

Treatments for these patients are largely anecdotal and have not been shown to improve or reverse the gangrene. Every effort should be made to identify and treat the underlying cause [3]. Early recognition of SPG, hemodynamic stability using intravenous fluids, and management of DIC are central to management [2,4]. Vasopressors should be avoided as they can worsen this condition. Repletion of coagulation factors or use of anticoagulants, based on whether bleeding or thrombosis is predominant, can help [3]. Sympathetic blockades, vasodilators, such as intravenous nitroprussides, local or intravenous infusion of a-blockers (phentolamine, chlorpromazine), and intravenous infusion of prostaglandin (epoprostenol) may be helpful. Ultimately, amputation of the gangrenous area after the appearance of a line of demarcation may be required [2,3]. Our patient was managed for heart failure with inotropes and diuretics. We did not place him on vasodilators with fear for decompensation of heart failure.

### 7.5. Prognosis

Prognosis is poor with mortality in one-tenth [17] to one-third of affected patients [16,18]. Most deaths occurred within 5 to 21 days after the onset of gangrene [18]. Of surviving cases, amputation (auto-amputation or surgical amputation) was reported in 67% [18] and 89% [16]. Thus, the development of SPG is an ominous prognostic sign, particularly in the background of DIC.

## 8. Conclusions

SPG is associated with significant mortality and morbidity with an alarmingly high rate of amputation in survivors. Awareness of this condition and association with a low flow state can help diagnose this condition early with prompt treatment for early recovery.

## Figures and Tables

**Table 1:** Pertinent labs with reference ranges.

S.N.	Labs	Outside Referring Hospital or on Admission	Prior to Discharge	Normal Reference Ranges
1.	**Liver function test**			
a.	Bilirubin, total	35.0	9.0	0.1–0.9 mg/dL
b.	Bilirubin, direct	>10.0	6.2	0.0–0.3 mg/dL
c.	Aspartate aminotransferase	309	192	7–42 IU/L
d.	Alanine aminotransferase	295	172	<45 IU/L
e.	Alkaline phosphatase	120	237	25–120 IU/L
f.	Albumin	3.0	2.6	3.2–4.9 g/dL
g.	Ammonia	39		11–35 µmol/L
2.	**Coagulation panel**			
a.	International normalized ratio	1.37	1.30	0.81–1.19
b.	Prothrombin time	15.2	14.4	8.9–13.1 sec
c.	Partial thromboplastin time	30	31	24–35 sec
d.	Platelet count	229		140–400 10^9^/L
3.	**Renal function test**			
a.	Sodium	131		135–146 mmol/L
b.	Potassium	4.0		3.3–4.8 mmol/L
c.	Chloride	97		89–109 mmol/L
d.	Bicarbonate	20		24–32 mmol/L
e.	Blood urea nitrogen	23		10–26 mg/dL
f.	Creatinine	0.9		0.7–1.7 mg/dL
4.	**Workup for etiology**			
a.	HBsAg	Negative		Negative
b.	HBCAb	Negative		Negative
c.	Hep A, IgM	Negative		Negative
d.	HCV Ab	Negative		Negative
e.	HIV Ab/p24 Ag	Negative		Negative
f.	HSV IgM	Negative		Negative
g.	VZV IgM	Negative		Negative
h.	Alcohol	<0.01		<0.01 g/dL
i.	Acetaminophen	Negative		Negative
j.	Anti-mitochondrial antibody	Negative		Negative
k.	Liver kidney microsomal antibody	Negative		Negative
l.	Anti-smooth muscle antibody	Negative		Negative
m.	Ceruloplasmin	Negative		Negative
